# The effect of vitamin D supplementation on the progression of fibrosis in patients with chronic liver disease: A protocol for a systematic review and meta-analysis

**DOI:** 10.1097/MD.0000000000020296

**Published:** 2020-05-08

**Authors:** Tiantian Chen, Xiaohong Zuo, Shengju Wang, Penglong Yu, Jie Yuan, Shujun Wei, Jiayi Chen, Yue Sun, Yongxiang Gao, Xueping Li

**Affiliations:** a Department of Rheumatology, Hospital of Chengdu University of Traditional Chinese Medicine, Chengdu 610072; b School of basic medical sciences, Chengdu University of Traditional Chinese Medicine, Chengdu 611137; c Department of Endocrinology, Hospital of Chengdu University of Traditional Chinese Medicine, Chengdu 610072; d College of International Education of Chengdu University of Traditional Chinese Medicine, PR China.

**Keywords:** chronic liver disease, hepatic fibrosis, meta-analysis, protocol, vitamin D supplementation

## Abstract

**Background:**

Hepatic fibrosis (HF) is the common pathological basis of chronic liver disease (CLD). Many data indicate that serum vitamin D (VD) levels in patients with liver fibrosis are significantly lower than those without liver fibrosis, and lower level of serum 1,25(OH)_2_D_3_ is also an independent risk factor for patients with liver fibrosis combined with other diseases. VD has the functions of anti-fibrosis, regulating cell proliferation and differentiation, anti-inflammatory, and immune regulation, Therefore, serum 1,25(OH)_2_D_3_ level may be negatively correlated with the progression of liver fibrosis. But there is absent convincing evidence-based medicine to confirm the efficacy of VD supplementation for CLD. Thus, we aimed to conduct this meta-analysis to summarize the efficacy of VD supplementation on the progression of fibrosis in patients with CLD.

**Methods:**

The study only selects clinical randomized controlled trials of VD supplementation for CLD. We will search each database from the built-in until September 2020. The English literature mainly searches Cochrane Library, Pubmed, EMBASE, and Web of Science. While the Chinese literature comes from CNKI, CBM, VIP, and Wangfang database. Meanwhile, we will retrieve clinical trial registries and gray literature. Two researchers worked independently on literature selection, data extraction and quality assessment. The dichotomous data is represented by relative risk (RR), and the continuous is expressed by mean difference (MD) or standard mean difference (SMD), eventually the data is synthesized using a fixed effect model (FEM) or a random effect model (REM) depending on the heterogeneity. The serum VD level, hepatic function and serological indexes of hepatic fibrosis were evaluated as the main outcomes. While several secondary outcomes were also evaluated in this study. The statistical analysis of this Meta-analysis was conducted by RevMan software version 5.3.

**Results:**

This meta-analysis will further determine the beneficial efficacy of VD supplementation on the progression of fibrosis in patients with CLD.

**Conclusion:**

This study determines the positive efficacy of VD supplementation for CLD.

**Ethics and dissemination:**

This review is based solely on a secondary study of published literatures and does not require ethics committee approval. Its conclusion will be disseminated in conference papers, magazines or peer-reviewed journals.

**Registration number:**

INPLASY202040054.

## 1 Introduction

Hepatic fibrosis (HF) is a pathological process in which extracellular matrix (ECM) is deposited in the liver in large amounts.^[1]^ HF is the common pathological basis of all chronic liver disease (CLD), and it is also a key step in the development of various CLDs to cirrhosis and an important link that affects the prognosis of CLD.^[1,2]^ Many data indicate that serum vitamin D (VD) levels in patients with liver fibrosis are significantly lower than those without liver fibrosis,^[3,4]^ and lower level of serum 1,25(OH)_2_D_3_ is an independent risk factor for patients with liver fibrosis combined with other diseases, including ischemic heart disease, malignant neoplasms, cirrhosis of the liver, ischemic stroke, lower respiratory tract infections, preterm birth complications, and diabetes mellitus,^[5]^ etc.

At present, it is widely believed that the transformation of hepatic stellate cells (HSCs) into myofibroblasts (MFLC) and fibroblasts is the central link in the development and development of HF.^[6]^ Oxidative stress and inflammation are starting factors.^[7–10]^ Liver fibrosis further develops into liver cirrhosis, even liver failure, and liver cancer. Common diseases that cause liver fibrosis include viral hepatitis (mainly hepatitis B and C), alcoholic liver disease, fatty liver disease (non-alcoholic fatty liver disease, NAFLD), autoimmune liver disease, cholestasis, parasitic infection, etc. Viral hepatitis is the most important cause of liver fibrosis in China, especially hepatitis B and hepatitis C. However, the common causes of liver fibrosis in developed countries in Europe and America are alcoholic liver disease because of excessive alcohol intake. NAFLD is the most common and emerging form of CLD worldwide, and almost 1/3 of NAFLD evolves in non-alcoholic steatohepatitis (NASH).^[11]^ NAFLD includes a wide spectrum of liver diseases ranging from simple fatty liver to steatohepatitis.^[12]^ Fibrosis is histologically reversible, and if active treatment is given during this period, liver fibrosis can be reversed.^[13,14]^ At present, there is no specific and effective treatment for fibrosis. The treatment methods mainly include the treatment of primary disease and anti-fibrosis treatment.

VD, including VD2 and VD3, is a group of biologically active fat-soluble steroid derivatives.^[15]^ 1,25-Dihydroxvitamin D3 [1,25(OH)_2_D_3_] is the hormonally active form of VD.^[16]^ In addition to its main role in regulating bone metabolism and calcium homeostasis,^[17]^ it has the functions of anti-fibrosis, regulating cell proliferation and differentiation, anti-inflammatory, and immune regulation,^[18–21]^ etc. VD plays a protective role in the progress of liver fibrosis, and it has an anti-fibrotic effect on hepatic stellate cells through a specific signal transduction pathway mediated by VD receptors.^[22–24]^

Many patients with CLD and animal models of liver cirrhosis, it is manifested that 25(OH)D_3_ levels are significantly reduced, and were significantly negatively correlated with liver fibrosis^[25,26]^ and liver function indicators, suggesting that 25(OH)D_3_ may be a protective factor for CLD.^[5,18]^ In the CCl_4_ mouse model, supplementation of 1,25(OH)_2_D_3_ can inhibit the proliferation and collagen secretion of HSC-T6 and HSC-T6 activated by NF-κB and TGF-β1 pathways, and lipogenesis and inflammation gene expressions were diminished, then ameliorate liver fibrosis and improve liver function.^[25,27–31]^ Some scholars have studied statistically significant improvements in metabolic indicators, oxidative stress, endothelial dysfunction and disease progression in NAFLD patients treated with silybin and VD and vitamin E, the proportion of which is greater than that without treatment patients with NAFLD.^[32]^ In conclusion, numerous data manifest that some serum 25(OH)D is closely related to the progress of liver fibrosis, and serum 1,25(OH)_2_D_3_ level may be negatively correlated with the progression of liver fibrosis. But there is absent convincing evidence-based medicine to confirm the efficacy of VD supplementation for CLD. However, further research is needed to fully elucidate its regulatory role in inhibiting liver fibrosis and to evaluate the safety and effectiveness of VD supplementation as a relatively inexpensive treatment for liver fibrosis in patients with CLD.

Meanwhile, based on numerous properties of VD, there has been a significant scientific interest in the relationship between VD status and CLD. We speculate VD supplementation is certainly beneficial for CLD patients with HF. Thus, we intend to collect randomized controlled trials (RCTs) about VD supplementation for CLD combined with HF based on evidence-based medicine and conduct a meta-analysis of its efficacy to provide higher quality clinical evidence for patients with CLD.

## 2 Methods

### 2.1 Protocol registration

The systematic review protocol has been registered on the INPLASY website (https://inplasy.com/inplasy-2020-4-0054/) and INPLASY registration number is INPLASY202040054. It is reported following the guidelines of Cochrane Handbook for Systematic Reviews of Interventions and the Preferred Reporting Items for Systematic Reviews and Meta-analysis Protocol (PRISM).^[33]^ If there are any adjustments throughout the study, we will fix and update the details in the final report.

### 2.2 Inclusion criteria

#### 2.2.1 Study design

The study only selects clinical RCTs of VD supplementation for CLD combined with HF published in both Chinese and English. However, animal experiments, reviews, case reports, and non-randomized controlled trials are excluded.

#### 2.2.2 Participants

The patients with clinically diagnosed CLD combined with HF and treatment with VD supplementation, regardless of race, gender, and age. HF by other causes and patients with severe heart disease, liver and kidney dysfunction, mental illness, or a relevant drug allergic history will be not included.

#### 2.2.3 Interventions

Both groups were treated with anti-fibrosis treatment and symptomatic treatment, including control of diet, moderate exercise, prohibition of drinking, antiviral and lipid-lowering therapies, etc. The experiment group used VD supplementation, while the control group applied for placebo, or no treatment. In addition, the two groups did not take any drugs that interfered with the outcome indicators. The follow-up time was ≥12 weeks.

#### 2.2.4 Outcomes

The primary outcomes include the improvement in clinical efficacy, serum VD level, hepatic function (ALT, AST), and hepatic indicators associated with fibrosis (HA, PC-III, C-IV, LN), blood test (PLT), calculate the APRI score and FIB-4 index based on the relevant indicators above.

Additional outcome(s): Secondary outcomes are mainly other indicators of liver function (GGT, TBIL, LDH, ALP, albumin, etc), coagulation test (APTT, PT, etc), blood calcium, blood phosphorus, portal vein inner diameter width and adverse events.

### 2.3 Search methods

#### 2.3.1 Electronic searches

Information sources: We will retrieve each database from the built-in until September 2020. The English literature mainly searches Cochrane Library, Pubmed, EMBASE, and Web of Science. While the Chinese literature comes from CNKI, CBM, VIP, and Wangfang database. We adopt the combination of heading terms and free words as search strategy, which decided by all the reviewers. Search terms: serum VD level, 25(OH)D, VD, 1,25(OH)_2_D_3_, VD deficiency, VD supplementation, nonalcoholic fatty liver disease, alcoholic fatty liver, steatohepatitis, liver fibrosis, liver cirrhosis, chronic hepatitis B, chronic hepatitis C, biliary atresia, primary biliary cirrhosis, schistosoma hepatic fibrosis, autoimmune hepatitis. We will simply present the search process of the cochrane library, as shown in Table 1, adjusting different search methods according to different Chinese and English databases.

**Table 1:**
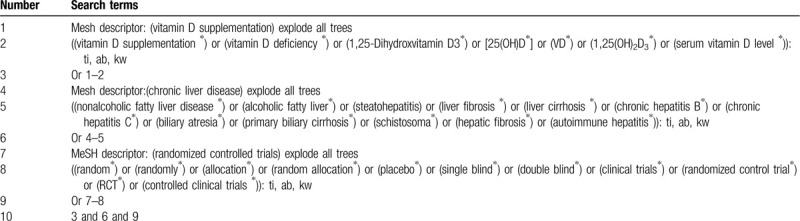
Example of Cochrane search strategy.

#### 2.3.2 Searching other resources

At the same time, we will retrieve other resources to complete the deficiencies of the electronic databases, mainly searching for the clinical trial registries and gray literature about VD with CLD on the corresponding website.

### 2.4 Data collection and analysis

#### 2.4.1 Selection of studies

**Figure 1:**
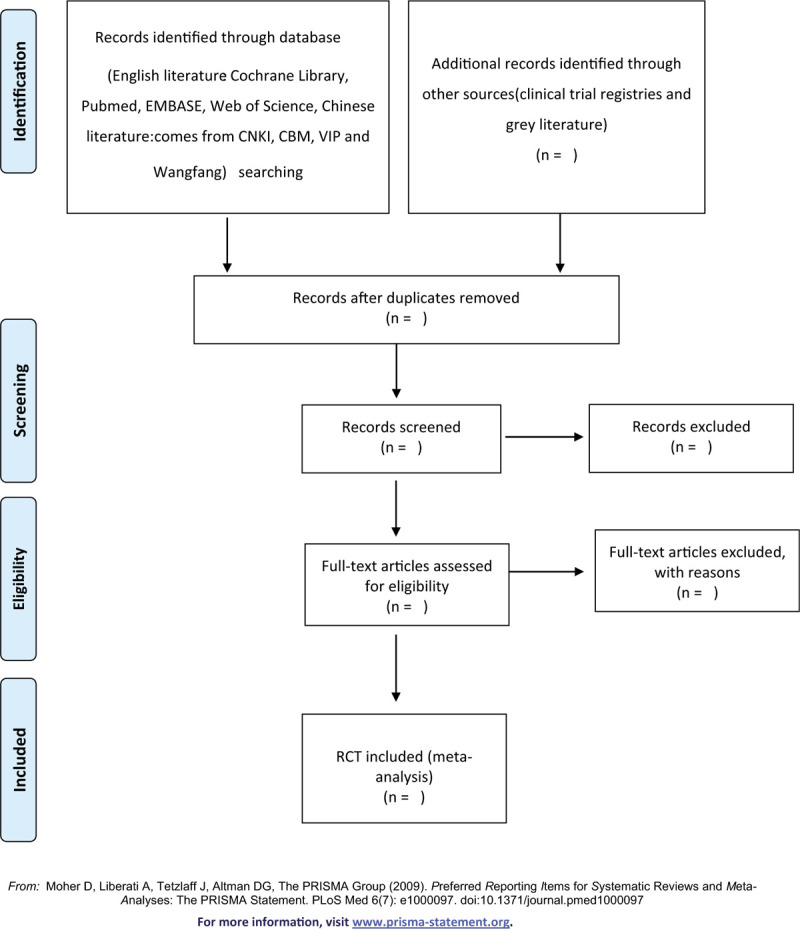
Flow chart of the study selection.

Import all literatures that meet the requirements into Endnote X8 software. Firstly, two independent reviewers initially screened the literatures that did not meet the pre-established standards of the study by reading the title and abstract. Secondly, download the remaining literatures and read the full text carefully to further decide whether to include or not. Finally, the results were cross-checked repeatedly by reviewers. If there is a disagreement in the above process, we can reach an agreement by discussing between both reviewers or seek an opinion from third party. PRISMA flow diagram (Fig. 1) will be used to show the screening process of the study.

#### 2.4.2 Data extraction and management

According to the characteristics of the study, we prepare an excel form for data collection before data extraction. Outcome indicators for eligible studies were independently extracted and filled in the data extraction form by two reviewers. If there is any argument, it can get an agreement by discussing through two reviewers or seek suggestions form third party. The main data extracted are as follows: title, author, year, fund source, sample size, age, gender, duration of disease, interventions, outcome measures, adverse reactions, etc. If you find something unclear in the study, you can contact the author of the communication directly for more detailed information. The above information was finally cross-checked by two reviewers.

#### 2.4.3 Assessment of risk of bias in included studies

The quality assessment of RCTs adopts the risk of bias (ROB) assessment tool provided by the *Cochrane Handbook*. The following seven items, such as random sequence generation, allocation concealment, blinding of participants and personnel, blinding of outcome assessment, incomplete outcome data, selective outcome reporting, and other bias, are evaluated by three grades of “low bias”, “high bias”, and “unclear bias”. The discrepancies will get a consistent conclusion by discussing between both reviewers or seeking the third-party consultation.

#### 2.4.4 Measures of treatment effect

Different evaluation methods are selected according to the different efficacy indicators. For the dichotomous data, we will choose the effect scale indicator relative risk (RR) with 95% confidence interval (CI) to represent. While the continuous data is expressed as mean difference (MD) or standardized mean difference (SMD) with 95% CI depending on whether the measurement scale is consistent or not.

#### 2.4.5 Dealing with missing data

The reviewers will contact the first author or correspondent author via email or telephone to obtain missing data if the relevant data is incomplete. If the missing data is still not obtained in the above way, we can synthesize the available data in the initial analysis. Furthermore, sensitivity analysis will be used to assess the potential impact of missing data on the overall results of the study.

#### 2.4.6 Assessment of heterogeneity

Heterogeneity will be assessed by Chi-squared test and *I*^2^ test. If *I*^2^ < 50%, *P* > .1, we consider that no statistical heterogeneity between each study and choose fixed effect model (FEM) to synthesize the data. If *I*^2^ ≥ 50%, *P* < .1, indicating that there is a statistical heterogeneity, the data is integrated by the random effect model (REM). In addition, due to differences in heterogeneity, we will conduct subgroup or sensitivity analysis to look for the potential causes.

#### 2.4.7 Data analysis

Review Manager software version 5.3 provided by the Cochrane Collaboration will be performed for data synthesis and analysis. The dichotomous data is represented by RR, continuous data is expressed by MD or SMD. If there is no heterogeneity (*I*^2^ < 50%, *P* > .1), the data is synthesized using a FEM. Otherwise (*I*^2^ ≥ 50%, *P *< .1), a random effect model is used to analyze. Then subgroup analysis will be conducted basing on the different causes of heterogeneity. If a meta-analysis cannot be performed, it will be replaced by a general descriptive analysis.

#### 2.4.8 Subgroup analysis

If the results of the study are heterogeneous, we will conduct a subgroup analysis for different reasons. Heterogeneity is manifested in the following several aspects, such as race, age, gender, different intervention forms, pharmaceutical dosage, and treatment course.

#### 2.4.9 Sensitivity analysis

Sensitivity analysis is mainly used to evaluate the robustness of the primary outcome measures. The method is that removing the low-level quality study one by one and then merges the data to assess the impact of sample size, study quality, statistical method, and missing data on results of meta-analysis.

#### 2.4.10 Grading the quality of evidence

In this systematic review, the quality of evidence for the entire study is assessed using the “Grades of Recommendations Assessment, Development and Evaluation (GRADE)” standard established by the World Health Organization and international organizations.^[34]^ To achieve transparency and simplification, the GRADE system divides the quality of evidence into four levels: high, medium, low, and very low.

## 3 Discussion

VD is becoming increasingly accepted as an important physiological regulator outside of its classical role in skeletal homeostasis. A growing body of evidence connects VD with hepatic diseases, and the therapeutic potential of VD-based treatments to protect against hepatic disease progression and to improve response to treatment.^[35]^ Nowadays, fibrosis lacks specific medicines. In the prevention and treatment of liver disease, the treatment of liver fibrosis determines the development of the disease. In the past few years, a large number of researches has been assembled that VD/VDR system attributes an important role in fibrosis,^[15,36]^ inflammation and hepatic aberrant fat accumulation,^[30,37]^ But there is absent convincing evidence-based medicine to confirm the efficacy of VD supplementation for CLD combined with HF. Thus, we attempt to conduct this meta-analysis to analysis and summarize the efficacy of VD supplementation for CLD combined with HF.

There are strengths in our study. Firstly, this meta-analysis provides a comprehensive assessment to whether VD supplementation is beneficial for CLD patients combined with HF. Secondly, this study will provide clear evidence that VD supplementation is good for CLD patients combined with HF. Moreover, RCTs will be included in our studies appear to be high quality and low risk of bias. However, there may be some limitations in our meta-analysis. At last, both Chinese and English forms of research may increase the bias of the study. Secondly, the variety of race, age, gender, intervention forms, pharmaceutical dosage and treatment course may result in higher clinical and statistical heterogeneity.

In conclusion, this study will help to determine the beneficial effects on CLD patients combined with HF. We hope this study will provide higher quality evidence for the benefits of VD supplementation for CLD combined with HF.

## Author contributions

**Conceptualization:** Tiantian Chen, Xiaohong Zuo.

**Data curation:** Tiantian Chen, Shengju Wang, Jiayi Chen.

**Formal analysis:** Penglong Yu, Jie Yuan, Yue Sun.

**Funding acquisition:** Yongxiang Gao, Xueping LI.

**Methodology:** Jie Yuan, Shujun Wei.

**Project administration:** Yongxiang Gao, Xueping LI.

**Resources:** Tiantian Chen, Shujun Wei.

**Software:** Tiantian Chen, Shengju Wang, Penglong Yu.

**Supervision:** Yongxiang Gao.

**Writing – original draft:** Tiantian Chen, Shengju Wang.

**Writing – review & editing:** Xueping LI.
